# Generation of Organized Porcine Testicular Organoids in Solubilized Hydrogels from Decellularized Extracellular Matrix

**DOI:** 10.3390/ijms20215476

**Published:** 2019-11-03

**Authors:** Maxime Vermeulen, Federico Del Vento, Marc Kanbar, Sébastien Pyr dit Ruys, Didier Vertommen, Jonathan Poels, Christine Wyns

**Affiliations:** 1 Gynecology-Andrology Research Unit, Institut de Recherche Expérimentale et Clinique, Medical School, Université Catholique de Louvain, 1200 Brussels, Belgium; federico.delvento@uclouvain.be (F.D.V.); marc.kanbar@uclouvain.be (M.K.); 2 Phosphorylation—MassProt Unit, Institut de Duve, Université Catholique de Louvain, 1200 Brussels, Belgium; sebastien.pyrditruys@uclouvain.be (S.P.d.R.); didier.vertommen@uclouvain.be (D.V.); 3 Department of Gynecology-Andrology, Cliniques Universitaires Saint-Luc, 1200 Brussels, Belgium; jonathan.poels@uclouvain.be

**Keywords:** decellularization, extracellular matrix, immature testicular tissue, three-dimensional culture, testicular cells, organoids, fertility preservation, artificial testis, spermatogonial stem cells

## Abstract

Cryopreservation of immature testicular tissue (ITT) prior to chemo/radiotherapy is now ethically accepted and is currently the only way to preserve fertility of prepubertal boys about to undergo cancer therapies. So far, three-dimensional culture of testicular cells isolated from prepubertal human testicular tissue was neither efficient nor reproducible to obtain mature spermatozoa, and ITT transplantation is not a safe option when there is a risk of cancer cell contamination of the testis. Hence, generation of testicular organoids (TOs) after cell selection is a novel strategy aimed at restoring fertility in these patients. Here, we created TOs using hydrogels developed from decellularized porcine ITT and compared cell numbers, organization and function to TOs generated in collagen only hydrogel. Organotypic culture of porcine ITT was used as a control. Rheological and mass spectrometry analyses of both hydrogels highlighted differences in terms of extracellular matrix stiffness and composition, respectively. Sertoli cells (SCs) and germ cells (GCs) assembled into seminiferous tubule-like structures delimited by a basement membrane while Leydig cells (LCs) and peritubular cells localized outside. TOs were maintained for 45 days in culture and secreted stem cell factor and testosterone demonstrating functionality of SCs and LCs, respectively. In both TOs GC numbers decreased and SC numbers increased. However, LC numbers decreased significantly in the collagen hydrogel TOs (*p* < 0.05) suggesting a better preservation of growth factors within TOs developed from decellularized ITT and thus a better potential to restore the reproductive capacity.

## 1. Introduction

Improvements of cancer therapy now allow more than 80% of children to survive their disease in Europe [1]. Unfortunately, chemotherapy and radiotherapy are associated with side effects, including gonadotoxicity, pointing to the need to develop fertility preservation methods [2]. If in adult men cryopreservation of a sperm sample before the start of gonadotoxic treatment is routinely proposed, this is not an option for prepubertal boys for whom cryopreservation of immature testicular tissue (ITT) containing spermatogonial stem cells (SSCs) is the only available method to preserve their fertility. A significant number of centers worldwide now offer this option [3,4,5]. In the case of non-hematological cancer or benign disease, autologous transplantation of ITT could be a promising method to restore fertility of patient from cryopreserved ITT [6,7]. Importantly, the power of this technique was recently demonstrated in monkey, with offspring generation following ITT autotransplantation [8]. In the context of cancer carrying the risk of neoplastic cell contamination of the testes, in vitro maturation and transplantation of a selected population of germ cells (GCs) including SSCs are so far the two approaches that may be considered to restore fertility using cryostored ITT [3]. In vitro maturation of ITT followed by successful generation of offspring were reported in mice [9] but in humans only generation of haploid GCs was achieved although with a very low efficiency [10,11]. Transplantation of isolated SSCs into seminiferous tubules also gave promising results in animals with offspring generation in several species [12,13,14,15,16] and embryo formation in monkey [17]. However, it has been reported that not only the GCs but also the somatic compartment of the testes, the so-called SSC niche, may be damaged by cancer therapies [18,19], which could hamper the success of SSC transplantation. In this regard, testicular organoids (TOs) developed with selected germ and somatic cells isolated from cryopreserved ITT with subsequent reconstitution of the SSC niche that could be transplanted back to the patient, represents a promising novel approach to restore fertility with cryostored ITT.

Formation of a functional organoid relies on the capacity of the cells to self-assemble in a three-dimensional structure showing organization and functions similar to those of the organ they mimic but also on exogenous components such as differentiation factors and a supportive testicular extracellular matrix (ECM) [20]. In the testis, spermatogenesis takes place in seminiferous tubules, containing GCs and Sertoli cells (SCs), which are surrounded by peritubular cells and the interstitial compartment where Leydig cells (LCs) produce testosterone. Testicular ECM components such as fibronectin and laminin produced by SCs and peritubular cells, respectively, as well as proteoglycans and collagen IV produced by both cell types, play an important role in spermatogenesis [21,22]. Indeed, abnormal localization of laminin and collagen were associated with Sertoli cell-only syndrome, cryptorchidism and testicular atrophy [23,24].

Moreover, the importance of the ECM for testicular cell suspensions (TCSs) organization in vitro was demonstrated in rats where TCSs were shown to form cords harboring mature SCs and partially differentiated GCs in ECM extracts but not onto plastic [25]. Three-dimensional culture of TCSs in natural ECM extracts such as Matrigel® and collagen was performed in mice [26,27] and rats [25,28,29,30] and generation of TOs whose structure and functions resemble that observed in vivo was only recently achieved in rodents [26,29]. To keep advantage of the testicular ECM, culture of human TCS onto decellularized testicular tissue scaffolds was also investigated. However, while the functionality of the seeded cells was demonstrated, either a proper cells organization was lacking [31] or the migration was reached but without proper cellular rearrangement [32]. Moreover, incubation of adult human TCSs in low attachment plates with medium containing testis-derived ECM resulted in their reaggregation into spheroids with GCs localizing in the center while peritubular and SCs surrounded the spheroids [33].

Furthermore, attempts to form TOs with cells from large animals (pigs and non-human primates) and human also led to cellular reaggregation with an abnormal organization as GCs and SCs were found in the outer part while LCs and peritubular cells located inside the TOs suggesting that TCSs cannot rebuild seminiferous tubule-like (ST-like) structures in low attachment plates [34].

Succeeding in TOs formation with TCSs isolated from large animals could bring valuable knowledge for development of human TOs that may further be used to unravel the physiopathology of reproductive disorders in the male and to develop a novel fertility restoration option in cases where transplantation of cryostored ITT is not a safe option. Accordingly, as pig and human share common properties at the genetic and physiologic level it makes porcine tissues or organs ideal models for human disease and xenotransplantation studies [35]. To preserve advantages of the testicular ECM (tECM) composition while facilitating the reorganization of TCSs in a hydrogel, decellularized porcine ITT scaffolds were solubilized and used for TOs formation. TOs formed in tECM were compared to TOs generated in collagen and to ITT cultured in a conventional organotypic system.

## 2. Results

### 2.1. Evaluation of Hydrogels

**Figure 1:**
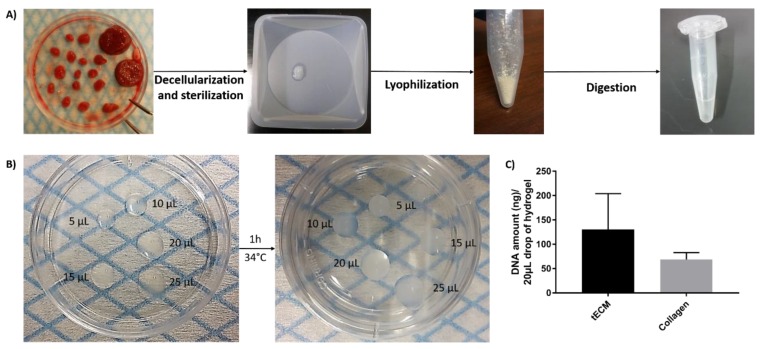
Formation of testicular extracellular matrix (tECM) hydrogel. (**A**) Porcine immature testicular tissues (ITTs) were dissected in small fragments and decellularized before being lyophilized and digested in a solution of HCl/pepsin (*n* = 20). (**B**) Drops of 5, 10, 15, 20 and 25 μL were incubated for 1 h at 34 °C to evaluate manipulability after gelation. (**C**) DNA amount/20 μL of tECM and collagen.

Solubilized tECM was produced by digestion of decellularized ITT fragments and drops of different sizes were used to evaluate manipulability after gelation (Figure 1A,B). Extracted deoxyribonucleic acid (DNA) was quantified and similar levels were found in tECM (130.7 ± 73.3 ng/20 μL drop) and control collagen (68.9 ± 14.0 ng/20 μL drop) hydrogels (Figure 1C). 

Analysis of tECM and collagen by two-dimensional liquid chromatography-tandem mass spectrometry (2D-LC-MS/MS) resulted in identification of 2176 and 63 proteins, respectively. Among the identified ECM-proteins, 41 were found in both hydrogels (Table S1). The collagen hydrogel was highly enriched in collagen type I but contained also other types of collagen (II, III, V and VI) in smaller amounts. However, tECM hydrogel was essentially composed of collagen types I, IV, VI, XII and XIV but also contained types II, III, V, VII, X, XV, XVIII and XXVII. Moreover, only one kind of ECM-glycoprotein was identified in the collagen hydrogel while tECM hydrogel contained more than 20 ECM-glycoproteins among whose fibronectins and laminins were the most abundant. Additionally, 13 proteoglycans were identified in tECM but none were present in the collagen hydrogel.

**Figure 2:**
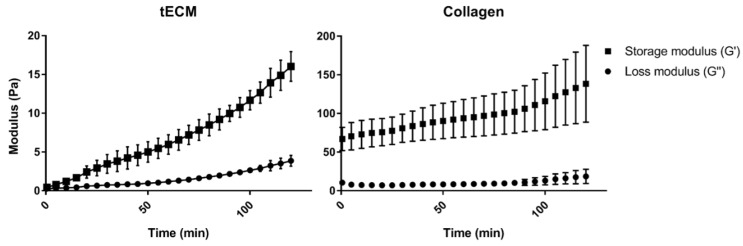
Rheological properties of hydrogels. Gelation kinetics were determined by monitoring of variations of storage (G′) and loss (G″) moduli. Data represented means ± standard deviation. *n* = 3.

Rheological analysis showed a higher storage modulus (G′) for the collagen hydrogel compared to tECM hydrogel (Figure 2). However, each hydrogel had a higher G′ than the loss modulus (G″) suggesting a solid-like property of both hydrogels.

### 2.2. Characterization of ITT-Isolated Cells

**Figure 3:**
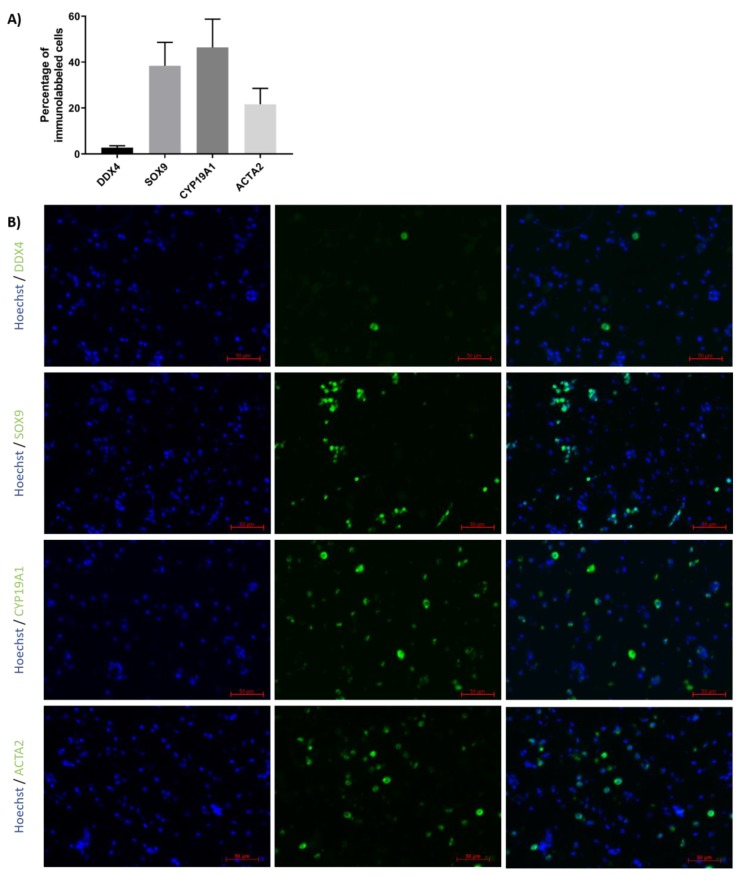
Percentage of different testicular cell types in testicular cell suspension (TCS). (**A**) The graph represents the percentage of germ cells (GCs; DDX4), Sertoli cells (SCs; SOX9), Leydig cells (LCs; CYP19A1) and peritubular cells (ACTA2) in TCS obtained following digestion of ITT and used for testicular organoid (TO) generation. (**B**) Representative image of DDX4, SOX9, CYP19A1 and ACTA2 immunofluorescence analysis of TCS used for generation of TOs. *n* = 4.

Immunofluorescence detection was performed to evaluate the proportions of GCs, SCs, peritubular cells and LCs in TCSs isolated from ITTs before generation of TOs (Figure 3A,B). Results revealed that 2.7 ± 0.9% expressed the GC marker DDX4, 38.4 ± 10.2% expressed the SC marker SOX9, 46.4 ± 12.3% expressed the LC marker CYP19A1 and 21.7 ± 6.9% expressed the peritubular cell marker ACTA2.

### 2.3. Evaluation of Porcine TO Organization

**Figure 4:**
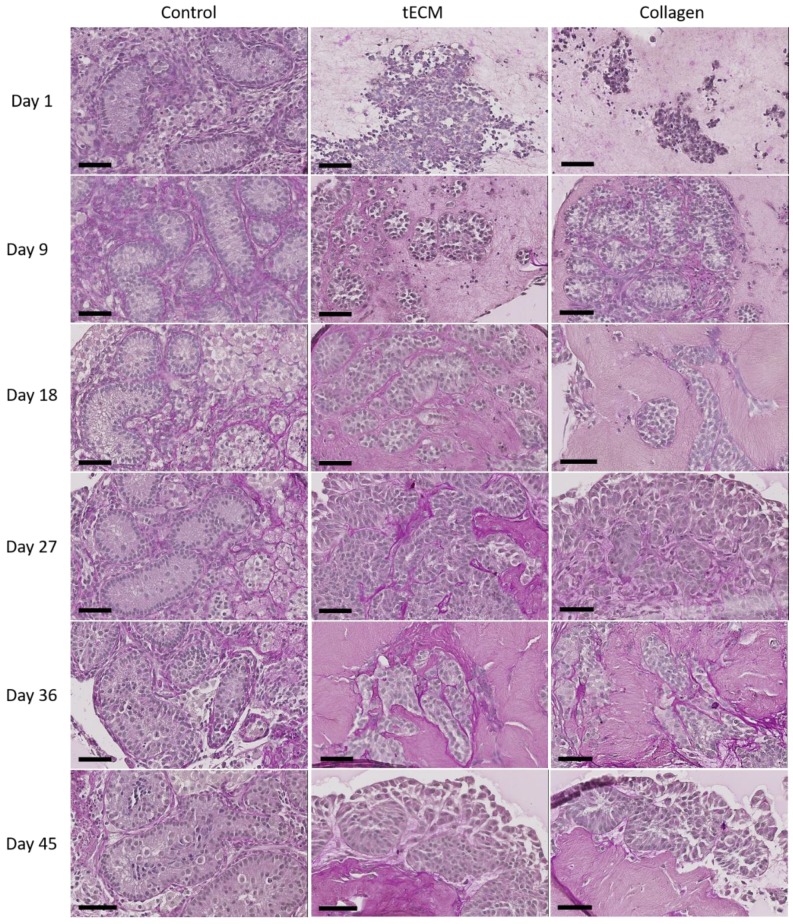
Periodic acid Schiff staining of control tissue and TOs formed in tECM and collagen during the culture period. Scale bars = 60 μm.

Periodic acid Schiff performed after 1 day of culture revealed no organization of testicular cells in ECM hydrogels. Figure 4 shows that ST-like structures surrounded by a basement membrane appeared in both ECMs during the nine first days of culture and were maintained until the end of the culture.

**Figure 5:**
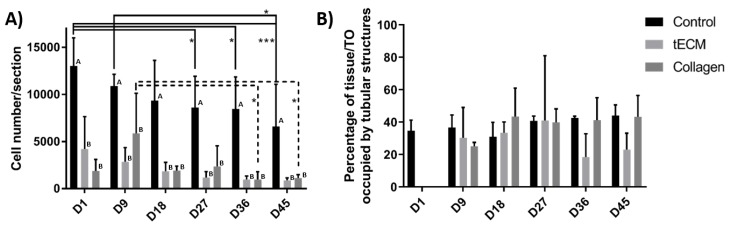
Control tissues and TOs characterization. (**A**) Number of cells per section. (**B**) Percentage of control tissue or TO occupied by tubular structures. *n* = 4, * *p* < 0.05, *** *p* < 0.001. Different letters represent significant differences (*p* < 0.05) between groups at each time point.

The total number of cells per section was significantly higher in the control group at each time point of the culture but did not show significant variations between the tECM and collagen groups at any time point (Figure 5A). However, cell numbers/section decreased significantly over time in control and collagen groups (Figure 5A). Percentage of area occupied by tubular structures found in control and TOs groups was also quantified (excepted in hydrogel groups on day 1 as ST-like structures were not yet formed) and remained stable in each group from day 9 to the end of the culture (Figure 5B).

**Figure 6:**
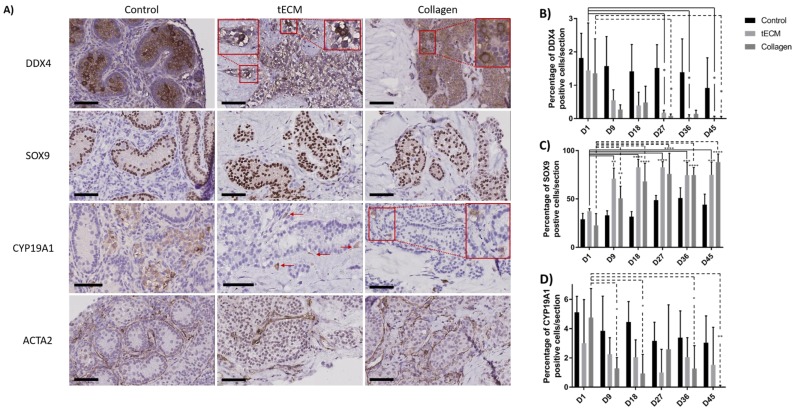
Identification and quantification of the different testicular cell types in control tissue and TOs. (**A**) Immunohistochemical analysis showing GCs (DDX4), SCs (SOX9), LCs (CYP19A1) and peritubular cells (ACTA2) in control tissue and TOs on the 9th day of culture. Quantification of GCs (**B**), SCs (**C**) and LCs (**D**) over the culture period in control tissue and TOs. *n* = 4, * *p* < 0.05, ** *p* < 0.01, *** *p* < 0.001, **** *p* < 0.0001. Scale bars = 60 μm.

Identification of GCs, SCs, LCs and peritubular cells was performed by immunohistochemistry (IHC) against DDX4, SOX9, CYP19A1 and ACTA2 antigens and demonstrated presence of all cell types during culture (Figure 6A). Moreover, DDX4 and SOX9 positive cells were localized inside ST-like structures while LCs and peritubular cells were observed outside. Rare CYP19A1-positive cells were identified inside tubular structures. Quantification of GCs, SCs and LCs was performed but this was not possible for peritubular cells due to the difficulty of discerning these cells from each other.

Numbers of DDX4+ cells were stable in the control group during the culture period. However, compared to day 1 of culture, numbers of DDX4+ cells were significantly lower in tECM group on day 27, 36 and 45 (*p* < 0.05) and in collagen group on day 27 and 45 (*p* < 0.05; Figure 6B).

SC numbers remained stable in the control group while they were significantly higher on day 9, 18, 27, 36 and 45 compared to day 1 of culture (*p* < 0.01) in tECM and collagen groups (Figure 6C).

With regard to LCs, the ratio between CYP19A1+ cells and total number of cells/section showed a significant decrease in the collagen group on day 9, 18, 36 and 45 (*p* < 0.05) compared to day 1. No significant differences were found in control and tECM groups (Figure 6D).

### 2.4. Functional Evaluation of Porcine TOs

**Figure 7:**
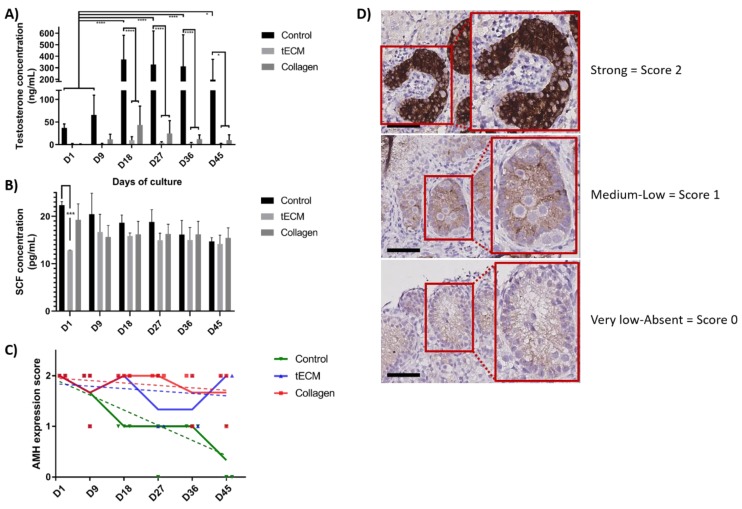
Evaluation of LC and SC functionality and maturation in control tissue and TOs. Testosterone (**A**) and stem cell factor (SCF) (**B**) were quantified in culture supernatants (*n* = 3). (**C**) Maturation of SCs was monitored in control tissue and TOs by immunohistochemistry (IHC) for anti-Mullerian hormone (AMH; *n* = 4). (**D**) Representation of the scores used to determine AMH intensity staining. * *p* < 0.05, *** *p* < 0.001, **** *p* < 0.0001. Scale bars = 60 μm.

The functionality of LCs evaluated by measurement of testosterone concentration showed a stable secretion in tECM and collagen TOs during the entire culture period but an increase in control group on day 18, 27, 36 and 45 (*p* < 0.05) compared to day 1 and 9. From day 18 until the end of the culture, testosterone secretion was significantly higher in the control group compared to tECM and collagen groups (*p* < 0.05; Figure 7A). However, testosterone secretion in tECM and collagen groups were not significantly different. Regarding SC functionality, stem cell factor (SCF) secretion was stable during the entire culture period and no differences were detected between the three groups except on day 1 for tECM compared to the control group (*p* < 0.001; Figure 7B). Maturation of SCs evaluated using a score based on anti-Mullerian hormone (AMH) immunostaining demonstrated a significant decrease over time in control (*p* < 0.001) but not in tECM and collagen groups (Figure 7C,D). 

### 2.5. Evaluation of GC Differentiation 

**Figure 8:**
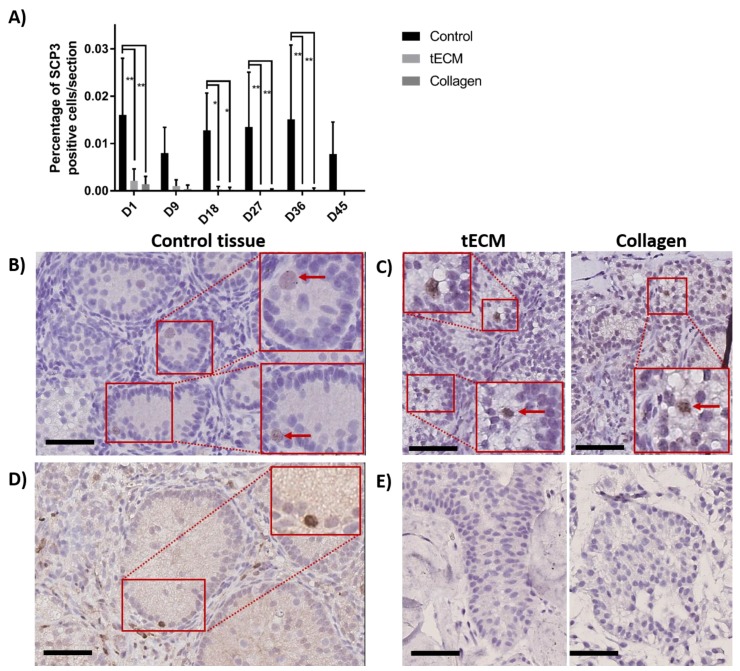
Evaluation of GC differentiation in control and TOs. (**A**) Number of SCP3 positive cells/section (*n* = 4). Weak staining was observed for SCP3 in 1/4 non-cultured ITTs on day 0. Detection of SCP3 in control (**B**) and TOs (**C**) on day 9 of culture. Detection of cAMP responsive element modulator (CREM) positive cells after 45 days of culture in control tissue (**D**) but not in TOs (**E**). * *p* < 0.05 and ** *p* < 0.01. Scale bars = 60 μm.

To detect meiotic differentiating GCs, IHC against synaptonemal complex protein 3 (SCP3) was performed on day 1, 9, 18, 27, 36 and 45 of culture. Figure 8A shows that the percentage of SCP3 positive cells/section in the control group was stable during the whole culture period while it decreased in TOs to reach zero at the end of the culture. Moreover, higher numbers of SCP3 positive cells/section were observed in control compared to tECM on day 1, 18, 27 and 36 (*p* < 0.05) and collagen on day 1, 18, 27, 36 and 45 (*p* < 0.05). Detection of SCP3 in control and TOs is showed in Figure 8B,C, respectively. Presence of differentiated GCs at the post-meiotic stage was evaluated by IHC against cAMP responsive element modulator (CREM) on controls and TOs and showed detection of the protein along the basement membrane only in 2/4 control tissues (Figure 8D) on day 45 of culture but not in TOs (Figure 8E). 

## 3. Discussion

There is a growing interest in three-dimensional organoid development notably for cancer modeling, high-throughput drug screening but also for the study of normal and pathological development of tissues/organs [36,37,38]. Here we reported for the first time, to the best of our knowledge, successful development of functional TOs in large mammals using ECM-based hydrogels.

Following decellularization of porcine ITT as described in previous work [31], acellular testicular scaffolds were digested with pepsin and used to produce a hydrogel (tECM). In this study, the tECM hydrogel was compared to a preparation of collagen, which previously allowed mouse ST-like structure formation and partial GC differentiation up to pachytene spermatocytes [26]. Since physical properties of the cell-supportive matrix were shown to modulate cellular expansion and organoid formation in vitro [39], rheological evaluation of our ECM hydrogels was performed and revealed a solid-like behavior of both hydrogels as their storage moduli were higher than their loss moduli [40]. Higher moduli observed for collagen hydrogel could be explained by the fact that collagen, as the most important ECM component for stabilization of hydrogels structure, and tECM were used at the same total protein concentration. This implies that the collagen amount was inevitably lower in tECM as we showed that it also contained other proteins that were shown to affect hydrogels mechanical properties, such as GAGs [41,42].

Several reports demonstrated that matrix stiffness modulation is key as it resulted in modification of primary [43] and stem cell [44] proliferation and differentiation. For instance, Matrigel® stiffness can be modulated by modification of its concentration and solutions of 4, 8 and 17 mg/mL resulted in storage moduli of 20, 70 and 300 Pa respectively [45]. Recently, Sun et al. reported differentiation of human spermatogonia isolated from testicular tissues of patients affected by obstructive azoospermia into haploid spermatids [46]. Interestingly, they used a 9 mg/mL solution of Matrigel® to cultivate mitotically inactivated SCs with GPR125^+^ spermatogonia in three dimensions and reported their differentiation into haploid cells after 20 days of culture. In their study, Alves-Lopes et al. used Matrigel® at a concentration of 4–6 mg/mL to constitute rat TOs that were maintained for 21 days in vitro, but unfortunately, differentiation was not evaluated [29]. In our study, storage modulus of tECM did not exceed 20 Pa so it would be interesting to produce TOs with tECM of different stiffnesses to evaluate the impact on TO culture outcomes, more specifically on GC differentiation. 

Components of the ECM are tissue-specific and constitute not only a structural support for cells but also act on their capacity to organize in three-dimensional structures [25,30,47,48]. Indeed, laminin was shown to promote cord-like structure in vitro [30] and both laminins and collagens are involved in GC migration from the basal lamina to the lumen of seminiferous tubules through regulation of restructuring events [49]. Therefore, it is relevant to hypothesize that formation of TOs by combination of a hydrogel containing important proteins of the testicular ECM with immature testicular cells could improve their reorganization and function.

As expected, analysis of protein content by 2D-LC–MS showed more collagen type I in the collagen hydrogel compared to the tECM hydrogel. However, collagens IV, VI, XII and XIV were more abundant in tECM hydrogel and other important ECM components such as fibronectin, laminin and nidogen-1 were not detected in the collagen hydrogel. GAGs are also known to be important components of the ECM as they were found to regulate the polymerization process of ECM-derived hydrogels, notably through their action on fibrils density [41]. Important proteoglycans (core protein attached to one or more GAG) were identified by 2D-LC–MS only in our tECM, notably, agrin and heparan sulfate, which were reported to be constituents of basement membranes [50,51] and decorin, which plays a role in collagen fibril assembly [52]. This greater similarity with the matrix composition found in vivo could be involved in the better preservation of LCs within the TOs produced with the tECM hydrogel.

One of the major features of organoids relies on the ability of seeded cells to reform organ-like structures [53]. In an interesting study, Yokonishi et al. demonstrated reformation of testicular structures in culture of immature mice testicular cells without the need of a 3D supportive matrix although germ cell differentiation beyond the meiotic phase was not observed [54]. However, when a 3D collagen supportive matrix was added in culture of immature rat cells, differentiation up to the spermatid stage was reached [28]. In their study, Baert et al. cultured human adult and peripubertal testicular cells onto decellularized testis scaffolds and reported maintenance of the major somatic cells of the testicular niche as well as proliferating GCs but did not observe a testis-specific cellular organization [32]. Furthermore, in one of our previous studies, we observed the inability of human adult SCs to migrate deeply into decellularized porcine ITT scaffolds [31]. These observations could be attributed to the developmental status of the cells, which may influence their morphogenic capacity. Indeed, so far only immature cells were shown to be able to reform testicular structures in vitro [29,55]. Here, we demonstrated formation and maintenance of ST-like structures by immature porcine TCSs from day 9 to 45 of culture in tECM and collagen hydrogels. Periodic acid Schiff allowed identification of a basement membrane around these structures and the different cell types reorganized themselves into testis-like structures similar to in vivo tissue organization as peritubular and LCs were located outside ST-like structures while SCs and GCs were observed inside the ST-like structures. However, some rare CYP19A1-labeled cells were found within the tubules that may correspond to rare gonocytes as it was demonstrated that some of them may still be present during the postnatal period [56]. To our knowledge, such seminiferous-like tubule reorganization was never achieved in large mammals.

During the culture, SC numbers/section were stable in control cultured tissue but increased in tECM and collagen groups. Immature SCs are characterized by a proliferative activity and a strong AMH expression until puberty followed by a down-regulation reflecting their terminal differentiation [57]. Analysis of AMH expression demonstrated SC maturation in control tissue but not in TOs, which could explain the higher number of SCs/section in TOs. The lack of SCs maturation in both TOs could result from the lower level of testosterone, which is involved in in situ SCs maturation [58]. However, functionality of SCs was confirmed throughout the culture as SCF was detected until the 45th day in both TOs except for a lower SCF level measured at the onset of culture in the tECM group. The latter observation is most likely due to an artifact due to supernatant mishandling as SCF secretion was not different from control tissue in all other culture time points. In future experiments, evaluation of expression of androgen receptor and proteins associated with the blood–testis barrier would give more information on the SCs maturation status [59].

In the prepubertal testis, SCs represent the major cell type and their proliferation is responsible for the volume increase of the gonad before puberty. Following puberty, testicular volume increases up to 15 fold mainly due to GCs proliferation making them the most abundant cell type in the testis [60]. In our study, the percentage of GC found in TCSs following tissue digestion was 2.7%, which is in accordance with the results of other researchers who found 1.6% of GC in TCSs isolated from prepubertal pigs [61]. During culture, numbers of GCs/section remained stable in control tissue while a progressive decrease was observed in both TOs reaching zero on day 45. This observation was also reported in the study of Alves-Lopes et al. who reported a reduction of the GC percentage/TO from 12% on day 7 to 4% on day 14 in rat TOs formed in Matrigel® [29]. Plausible explanations could be that GCs, which do not migrate into ST-like structures of TOs, enter apoptosis and/or induction of a cellular stress during the enzymatic digestion step. Due to the small number of GCs in TOs such evaluation was not performed in this experiment and needs to be further investigated.

The importance of testicular architecture for the mediation of SSC responsiveness to retinoic acid was recently underlined in mice [62]. As ST-like structures did not appear when complete medium (basic medium enriched with bone morphogenic protein 4 (BMP4), retinol, human chorionic gonadotropin (hCG) and follicle-stimulating hormone (FSH)) was added to TOs from the first day of culture (data not shown), basic medium composed of KnockOut™ Serum Replacement (KSR) and basic fibroblast growth factor (FGF2) was used during the first 8 days of culture to allow ST-like structures formation. Complete medium was thus used from day 9 onwards and as expected, induced a significant increase of testosterone levels in control tissue on day 18, 27, 36 and 45. The higher number of cells in control tissues compared to TOs could explain the higher testosterone concentration found in culture supernatants. However, testosterone secretion during the whole culture period as well as the small although non-significant increase observed in TO groups suggests LC responsiveness to hCG in TOs. Assessment of LC numbers/section showed a significant decrease of LCs only in collagen TOs suggesting a poorer LC preservation in this group. Presence of fibronectin only in the tECM hydrogel could be one explanation of this result as integrin-mediated adhesion to fibronectin was reported as a mitogen signal for LCs [63]. Nevertheless, the slight decrease observed in control and tECM groups suggest that it is still important to further improve the culture media composition.

To assess differentiation of GCs in control and TOs, IHC against meiotic (SCP3) and post (CREM) meiotic markers was performed and indicated stability of the percentage of SCP3 positive cells/section in control but progressive loss in both TOs. It may be assumed that the low number and the abnormal location of CREM-positive cells along the basement membrane in control tissues could be related to an incomplete establishment of the blood–testicular barrier that normally separates basal and adluminal compartments. This phenomenon was already observed in organotypic culture of human immature testicular tissue and could preclude the normal germ cell differentiation process including the migration towards the adluminal compartment [59].

Cell numbers/section and area occupied by tubular structures in control and TOs suggested absence of growth in vitro. Cell confinement could be one of the factors that may play a role and that should further be analyzed. Indeed, in vivo, a non-elastic fibrous membrane named albuginea is responsible for the interstitial pressure in the testis [64]. Experiments conducted in rat revealed the importance of the pressure exerted by albuginea as capsulotomy resulted in infertility 30 days after surgery [65] and several studies showed that growth and migration are modified when cells are placed in a confined environment [66,67]. Improvement of TOs oxygenation could also result in better culture outcomes as a recent study demonstrated a higher growth of ITT when cultured between an oxygen-permeable polydimethylsiloxane (PDMS) chip and an agar block partially immersed into medium compared to standard organotypic culture [68].

In conclusion, we demonstrated for the first time that TCSs isolated form porcine ITT could form TOs with seminiferous tubule organization comparable to the native organ when in vitro cultured in hydrogels. While both TOs showed somatic cell functionalities that were maintained until the end of the culture, tECM hydrogel allowed a higher number of LCs compared to collagen hydrogel. As GCs numbers decreased and SCs increased in both TOs and limited development of the TOs was achieved, further studies including modification of the medium composition, protein concentration of tECM and modulation of the pressure exerted onto TOs would be valuable.

## 4. Materials and Methods 

### 4.1. Decellularization of Porcine Immature Testicular Tissue

In accordance to the European directive for the protection of pigs (2008/120/EC), piglets intended for meat production can be castrated. Following castration, testes were placed in phosphate buffer saline (PBS) and transported to the lab at 4 °C. A total of 40 prepubertal testes recovered from castrated animals aged between 4 and 7 days old were decellularized using our previously established protocol [31]. Briefly, tissues were dissected in small fragments of ±5 mm^3^ and stored at −80 °C in PBS until use. Tissues were thawed in a water bath prewarmed at 37 °C, washed for 15 min in 2× PBS and agitated in 0.01% sodium dodecyl sulfate (SDS; VWR Chemicals, Leuven, Belgium, 33629.266) for 7 h followed by 1 h of agitation in 1% Triton X-100 (Sigma-Aldrich, Overijse, Belgium, X-100). During the decellularization process, rinse steps (5 min of agitation in deionized water + 15 min in 2× PBS) were performed on the 2nd, 4th, 6th and 7th hour before being rinsed in PBS and kept at 4°C in PBS. The next day, decellularized tissues were sterilized by agitation in 0.1% peracetic acid (Sigma-Aldrich, 77240)/4% ethanol (VWR, 20821.310) at 4 °C and rinsed three times in PBS (Lonza, Verviers, Belgium, BE17-516F).

### 4.2. Hydrogel Formation

Following lyophilization, tissues were mechanically powdered with scissors and digested for 24 h in 0.01 M HCl (Sigma-Aldrich, 1.00319) containing 2 mg/mL of pepsin (Sigma-Aldrich, P6887) in sterile 50 mL tubes (VWR, 734-0448). Digested tECM was centrifuged for 5 min at 1000 g to eliminate debris before neutralization of pH and salt concentration of the solution were realized by addition of one-tenth the digest volume of 0.1M NaOH (Sigma-Aldrich, 1.06462) and one-ninth the digest volume of 10× PBS (Sigma-Aldrich, D1408), respectively. Solubilized tECM was aliquoted and kept at −80 °C until use.

### 4.3. Evaluation of the Solubilized tECM and Collagen

#### 4.3.1. DNA Content Quantification

Total DNA was extracted from 200 μL of solubilized tECM and from collagen type I solution (Sopachem, Ghent, Belgium, 638-00661) both diluted at 2.4 mg/mL of protein using PureLink™genomic DNA mini kit (Thermo Fisher,Ghent, Belgium, K1820-01) following instructions of the manufacturer. DNA content was measured in triplicate with Nanodrop (Thermofisher).

#### 4.3.2. Rheological Analysis

Rheological characteristics of tECM and collagen were determined for three biological replicates using a parallel plate rheometer (Anton Paar, Graz, Austria, MCR102). Cold hydrogels were kept on ice and placed between two pre-cooled (4 °C) 25 mm plates separated by a 0.2 mm gap in a humidified chamber. Plates were progressively warmed to 34 °C and samples were subjected to an oscillatory strain of 1% at a constant angular frequency of 1 rad s^−1^ for two hours with measurements taken every 0.5 min.

#### 4.3.3. Mass Spectrometry Analysis

##### Sample Preparation for Mass Spectrometry Analysis

Total protein content was quantified by the BCA assay (Waltham, Massachusetts, USA). Five hundred µg of proteins were reduced and alkylated. Proteins were then precipitated by the use of a methanol/chloroform procedure, before being resuspended in 100 mM ammonium bicarbonate (Sigma Aldrich, MO, USA). Protein digestion was performed using trypsin (Promega, WI, USA). 

##### 2D-LC/MS

2D-LC–MS analysis was performed essentially as previously described [69]. Peptides were dissolved in solvent A (0.1% trifluoroacetic acid (TFA) in 2% ACN), directly loaded onto reversed-phase pre-column (Acclaim PepMap 100, Thermo Scientific, San Jose, CA) and eluted in backflush mode. Peptide separation was performed using a reversed-phase analytical column (Acclaim PepMap RSLC, 0.075 mm × 250 mm, Thermo Scientific) with a linear gradient of 4–36% solvent B (0.1% TFA in 98% ACN) for 36 min, 40–99% solvent B for 10 min and holding at 99% for the last 5 min at a constant flow rate of 300 nL/min on an Ultimate 3000 RSLN nanoHPLC system (Thermo Scientific). The peptides were analyzed by an Orbitrap Fusion Lumos tribrid mass spectrometer (Thermo Scientific). The peptides were subjected to nanospray ionization (NSI) source followed by tandem mass spectrometry (MS/MS) in Fusion Lumos coupled online to the ultra performance liquid chromatography. Intact peptides were detected in the Orbitrap at a resolution of 120,000. Peptides were selected for MS/MS using higher-energy collisional dissociation setting at 35 and collision-induced dissociation at 30 with multistage activation for neutral loss of phosphoric acid (97.976 Da); ion fragments were detected in the Iontrap. A data-dependent procedure of MS/MS scans was applied for the top precursor ions above a threshold ion count of 5.0E3 in the MS survey scan with 30.0s dynamic exclusion. The total cycle time was set to 4 s. Mass spectrometry level 1 spectra were obtained with an automatic gain control (AGC) target of 4E5 ions and a maximum injection time of 50 ms, and mass spectrometry level 2 spectra were acquired with an AGC target of 1E4 (10,000) ions and a maximum injection time of 35 ms. For MS scans, the mass over charge (m/z) scan range was 350 to 1800. The resulting MS/MS data was processed using Sequest HT search engine within Proteome Discoverer 2.2 (Thermo Fisher Scientific, MA, USA) against a human protein reference database obtained from Uniprot. Trypsin was specified as cleavage enzyme allowing up to two missed cleavages, four modifications per peptide and up to three charges. Mass error was set to 10 parts per million for precursor ions and 0.1 Da for fragment ions. Oxidation on methionine, phosphorylation on serine, threonin and tyrosine were considered as variable modifications. False discovery rate was assessed using Percolator (Thermo Fisher Scientific, MA, USA) and thresholds for protein, peptide and modification sites were specified at 1%.

### 4.4. Preparation and Evaluation of TOs

#### 4.4.1. Testicular Tissue Dissociation

Fresh testicular tissues recovered from castrated animals were transported to the lab at 4 °C in HBSS (Sigma-Aldrich, H9269) containing 10 units/mL of penicillin and 10 μg/mL of streptomycin (Thermofisher Scientific, Ghent, Belgium, 15140122) and dissected in small fragments. Tissues from four animals were digested in HBSS supplemented with 1 mg/mL of collagenase D (Sigma-Aldrich, 11088858001) and 1 mg/mL of DNase I (Sigma-Aldrich, 11284932001) for 20 min at 37 °C with repeated flushing every 3–4 min. Cellular suspensions were passed through a 70-μm cell strainer (VWR, 732-2758) and rinsed two times with HBSS to eliminate residual collagenase and DNase. Following counting with trypan blue and a Bürker chamber, testicular cells were used for preparation of TOs.

#### 4.4.2. Formation and Culture of TOs in tECM and Collagen

Collagen type I and tECM were prepared at a protein concentration of 2.4 mg/mL (maximum concentration that could be obtained after collagen reconstitution) and kept on ice to avoid gelation. To form TOs, 500,000 testicular cells were diluted in 200 μL of PBS and pelleted in the bottom of 1.5 mL Eppendorf tubes by centrifugation for 5 min at 400 *g* followed by elimination of PBS. Using pipette tips of 200 μL cut at about 1 cm from the pointed end, cell pellets were recovered with 20 μL of hydrogel and placed onto the lid of a petri dish (hanging drop method) filled with 2 mL of PBS for 1 h to allow gelation of hydrogel without spreading onto plastic. Organoids and control fresh ITT pieces of 1 mm^3^ were transferred onto culture inserts (Millicell® cell culture inserts, Merck, Overijse, Belgium, PICM01250) and cultured in 300 uL of DMEM/F12 (Thermo Fisher Scientific, 11330057) containing 10% of KSR (ThermoFisher Scientific, 12618013) as it was demonstrated to promote testicular structure formation from immature testicular cells [26], 10 ng/mL of FGF2 (R&D Systems, 233-FB-025) as it was shown to increase the growth of seminiferous cord in vitro [70] and favor self-renewal and production of committed progenitors [71] and 3 mg/mL of ceftazidime (Biopharma, Italy, Kefadim). Medium was changed every two days and enriched on the 9th day of culture with 100 ng/mL of BMP4 (ThermoFisher, PHC9534) as it stimulates c-kit expression [72], 1 μM of retinol (Sigma-Aldrich, R7632) for its effect on spermatid and spermatozoa numbers during in vitro maturation of ITT [73], 35 IU/L of FSH (Merck Serono, United Kingdom, Gonal F) for its protective role of germ cell protection against apoptosis [74] and 2 IU/L of hCG (MSD, the Netherlands, Pregnyl) for its LH-like action to stimulate testosterone secretion by Leydig cells [75] until the end of the culture.

#### 4.4.3. Histology and Immunohistochemistry/Immunofluorescence

Approximately 45 × 10^6^ testicular cells recovered from fresh ITT dissociation before generation of TOs were fixed in 4% paraformaldehyde (PFA; VWR, 9713) for 15 min then pelleted in 3 mL of 2% agar and embedded in paraffin prior to 5-μm sections preparation. Following deparaffinization and rehydration in toluene and alcohol baths, endogenous peroxidase activity was blocked in 0.3% H_2_O_2_ for 30 min followed by antigen retrieval in citrate buffer for 50 min at 98 °C. Nonspecific reaction was blocked with Tris-buffered saline (TBS) Tween 0.05% (TBST) containing 5% BSA (Sigma-Aldrich, A7030) for 30 min before primary antibodies (Table 1) diluted in TBST/1% BSA were added to samples for one night at 4 °C. The next day, secondary anti-rabbit or anti-mouse antibodies (Envision + system-labeled polymer-horseradish peroxidase; Agilent, Heverlee, Belgium, K4003 or K4001) were added for 40 min at room temperature (RT). Following washing in TBST, antibody signals were detected using Alexa Fluor™ 488 Tyramide (Thermofisher, B40953). Nuclei were counterstained using 10 μg of bisBenzimide H 33442 trihydrochloride (Sigma-Aldrich, 14533) diluted in TBST/10% BSA. After final washes in TBST and distilled water, slides were mounted using Dako fluorescence mounting medium (Agilent, S3023). Images were acquired on a Zeiss AxioImager.z1 (Zeiss, Germany) and quantification of nuclei and cells expressing DDX4, SOX9, CYP19A1 and ACTA was performed on three randomly selected fields/section using Fiji ImageJ software (National Institute of Health, USA, v1.47).

Control tissues and TOs were rinsed in PBS and fixed for 1 hour in 4% PFA at RT. Following alcohol dehydration, control tissues and TOs were immersed in xylene and embedded in paraffin for preparation of five μm-thick sections on Superfrost Plus slides (VWR, 631-0108). Sections underwent deparaffinization and rehydration before periodic acid Schiff staining or IHC. For IHC, endogenous peroxidase activity and antigen retrieval were performed as described in the previous paragraph. Slides were washed in 0.05 M TBS containing 0.05% of Triton X-100 (wash buffer) and incubated for 30 min in blocking solution composed of 10% normal goat serum (Thermofisher, 10000C) and 1% BSA. Primary antibodies (Table 1) diluted in 1% normal goat serum/0.1% BSA solution were added to the sections and incubated overnight at 4 °C in a humidified chamber. The next day, secondary anti-rabbit or anti-mouse antibodies were added for 60 min at RT. Diaminobenzidine (Agilent, K3468) was used as a chromogen. Following brief counterstaining with Mayer’s hematoxylin (Agilent, S3301), the slides were mounted with Dako mounting medium (Agilent, CS703). Slides were scanned with the Leica SCN400 scanner (Leica Biosystems, Wetzlar, Germany) and images were captured with the Aperio Imagescope software (Leica Biosystem, Vista, CA, USA). Positive and negative controls are presented in Figure S1.

Specific cell numbers/section were determined using the Fiji ImageJ software by calculating the percentage of cells stained for DDX4, SOX9 or CYP19A1/section and the total number of cells/section (= number of nuclei/section) using the following formula: (number of stained cells/section)/(total number of nuclei/section) × 100.

Area of tubular structure within tissues and TOs were determined with the Aperio Imagescope software. Percentage of tissue or TO occupied by tubular structures was then determined as follow: (total area occupied by tubular structures)/(total area of tissue or TO) × 100.

#### 4.4.4. Evaluation of LC and SC Functionality by ELISAs

Reagents of the SCF (Bio-Connect Diagnostics, The Nederland’s SEA120Po) and testosterone (VWR, ABNOKA2349) ELISA kits were prepared following manufacturer’s instructions. Supernatants from control, tECM and collagen groups were homogenized and centrifuged before use (*n* = 3). In the SCF assay, undiluted samples were used. For testosterone concentration evaluation, 150-fold dilution was necessary for supernatants of the control group while a five-fold dilution was applied to supernatants of the tECM and collagen groups. Final concentrations were calculated taking into account the dilution factors.

### 4.5. Statistical Analysis

Statistical analyses were performed with the GraphPad Prism 7 software (GraphPad Software, La Jolla, CA, USA). Data are presented as mean ± SD. All results were analyzed using a two-way ANOVA followed by a Tukey’s post-hoc test (*n* = 4 for IF, histologic and IHC analyses, *n* = 3 for ELISA measures). Scores relative to AMH intensity staining were subjected to a linear regression analysis (*n* = 4). Maturation of SCs was validated if the slope of line obtained for the linear regression was significantly non-zero indicating a decrease of the score over time.

## Figures and Tables

**Table 1:** Antibodies used in the study.

Antibody	Reference	Manufacturer	Dilution	Target Cell
ACTA2	A2547	Sigma-Aldrich	1:2000	Peritubular cells
AMH	MCA2246	Bio-Rad	1:800	Sertoli cells
CREM	Ab230543	Abcam	1:100	Spermatids
CYP19A1	Ab139492	Abcam	1:2000	Leydig cells
DDX4	Ab13840	Abcam	1:2000	Germ cells
SOX9	Ab185966	Abcam	1:2000	Sertoli cells
SCP3	HPA039635	Sigma-Aldrich	1:3000	Spermatocytes

## Supplementary Materials

Supplementary materials can be found at https://www.mdpi.com/1422-0067/20/21/5476/s1, Table S1: List of ECM proteins identified by 2D-LC-MS in tECM and collagen preparation. Proteins are classified alphabetically and different colors were used to categorize them: green = collagens, orange = ECM-glycoproteins, yellow = proteoglycans, blue = other ECM-affiliated proteins and grey = non ECM-affiliated proteins. *acession numbers = F1SGG3* and F1RGX4**. Sum posterior error probability (PEP) score = protein score calculated as the negative logarithms of the PEP values of the connected peptide spectrum matches (PSMs); Figure S1: IHC controls. Porcine ITT recovered from pigs aged between 4 and 7 days old was used as positive control for DDX4 (A), SOX9 (B), CYP19A1 (C) and ACTA2 (D) immunostainings. Positive staining for SCP3 and CREM were detected in mature (6 months old) porcine testicular tissue (F-H) but not in ITT (E-G). The expression of AMH was detected in STs of porcine ITT (I) but not in mature testicular tissue (J). Pictures E and F represent negative controls (without primary antibodies) with anti-mouse (E) and anti-rabbit (F) secondary antibodies. Scale bars = 50 μm.

## Abbreviations

2D-LC-MS	Two-dimensional liquid chromatography-tandem mass spectrometry
ACTA2	Actin alpha 2
AMH	Anti-Mullerian hormone
BMP4	Bone morphogenic protein 4
CREM	CAMP responsive element modulator
CYP19A1	Cytochrome P450 Family 19 Subfamily A Member 1
DDX4	DEAD-Box Helicase 4
ELISA	Enzyme-linked immunosorbent assay
FGF2	Fibroblast growth factor 2
FSH	Follicle-stimulating hormone
G′	Storage modulus
G″	Loss modulus
GAG	Glycosaminoglycan
GC	Germ cell
hCG	Human chorionic gonadotropin
IHC	Immunohistochemistry
ITT	Immature testicular tissue
KSR	Knock-out serum replacement
LC	Leydig cell
PBS	Phosphate-buffered saline
SC	Sertoli cell
SCF	Stem cell factor
SOX9	SRY-Box 9
SSC	Spermatogonial stem cell
SCP3	Synaptonemal complex protein 3
ST-like	Seminiferous tubule-like
TBS	Tris-buffered saline
TBST	TBS-Triton
TCS	Testicular cell suspension
tECM	Testicular extracellular matrix
TO	Testicular organoid
