# Supplementary material for: Generation of Organized Porcine Testicular Organoids in Solubilized Hydrogels from Decellularized Extracellular Matrix
Source: Int J Mol Sci. 2019 Nov 3;20(21):5476. doi: 10.3390/ijms20215476 (PMC6862040; doi:10.3390/ijms20215476)
Supplement: Supplementary file 1 [file ijms-20-05476-s001.pdf]

Supplemental Data

Table S1. List of ECM proteins identified by 2D-LC-MS in tECM and collagen preparation. Proteins are classified alphabetically and different colors were used to categorize them: green = collagens, orange = ECM-glycoproteins, yellow = proteoglycans, blue = other ECM-affiliated proteins and grey = non ECM-affiliated proteins. *acession numbers = F1SGG3* and F1RGX4**. Sum posterior error probability (PEP) score = protein score calculated as the negative logarithms of the PEP values of the connected peptide spectrum matches (PSMs).

	**Sum PEP score**			Sum PEP score	
**Protein name**	**tECM**	Collagen	Protein name	tECM	Collagen
Aggrecan core protein	10.3	/	Fibulin 5	3.6	/
Actinin alpha 4	6.3	2.5	Fibulin-1	5.2	/
ADAM metallopeptidase with thrombospondin type 1 motif 2	2.7	/	Galectin	1.8	3.2
ADAMTS like 4	1.6	/	Galectin	5.3	/
Agrin	35.9	/	Galectin	17.1	/
Apolipoprotein A-I	33.2	5.5	Heat shock protein HSP 90-alpha	5.5	2.1
Asporin	6.2	/	Hemoglobin subunit beta	17.0	5.6
ATP synthase subunit beta	56.8	5.0	Heparan sulfate proteoglycan 2	248.7	/
Biglycan	8.7	/	Histone H2B	17.8	5.2
Clathrin heavy chain	28.0	3.5	Histone H4	54.7	7.4
Collagen alpha-1(III) chain precursor	30.6	62.3	Hyaluronan and proteoglycan link protein 1	30.0	/
Collagen alpha-1(V) chain precursor	5.7	24.5	Junction plakoglobin	10.0	5.1
Collagen alpha-1(X) chain precursor	2.3	/	Keratin 10	18.1	5.6
Collagen alpha-2(I) chain precursor	128.5	665.9	Keratin 14	26.4	2.3
Collagen alpha-2(V) chain precursor	2.3	19.9	Keratin 5	24.0	6.7
Collagen alpha-3(V) chain precursor	3.7	/	Keratin 75	11.2	8.7
Collagen type I alpha 1 chain	94.0	849.8	Keratin, type II cytoskeletal 2 epidermal	12.0	6.4
Collagen type II alpha 1 chain	7.1	67.6	Laminin subunit alpha 1	50.4	/
Collagen type IV alpha 2 chain	44.6	/	Laminin subunit alpha 2	35.5	/
Collagen type IV alpha 5 chain	12.6	/	Laminin subunit alpha 3	10.5	/
Collagen type VI alpha 2 chain	12.3	/	Laminin subunit alpha 4	13.1	/
Collagen type VI alpha 3 chain	196.8	31.8	Laminin subunit alpha 5	91.9	/
Collagen type VI alpha 3 chain	159.2	/	Laminin subunit beta 1	32.7	/
Collagen type VI alpha 6 chain	4.9	/	Laminin subunit beta 2	47.6	/
Collagen type VII alpha 1 chain	2.7	/	Laminin subunit gamma 2	2.1	/

Collagen type XII alpha 1 chain	138.8	9.9	Laminin subunit gamma 3	2.3	/
Collagen type XIV alpha 1 chain	64.0	15.0	Laminin subunit gamma-1 precursor	56.7	2.3
Collagen type XIV alpha 1 chain	2.1	/	Lumican precursor	20.4	/
Collagen type XV alpha 1 chain	25.3	/	Matrilin 2	37.8	/
Collagen type XVIII alpha 1 chain	15.6	/	Matrix metalloproteinase-9 precursor	1.9	/
Collagen type XXVII alpha 1 chain	6.0	/	Multimerin 1	3.7	/
Cytochrome c	16.0	14.6	Multimerin 2	21.9	/
D-3-phosphoglycerate dehydrogenase	12.9	6.7	Myosin heavy chain 14	63.5	2.6
Decorin	10.7	/	Myosin light chain 1/3, skeletal muscle isoform	10.2	2.4
Dermatopontin	3.3	/	Nidogen 1	27.4	/
Dolichyl-diphosphooligosaccharide-protein glycosyltransferase subunit STT3A	32.1	2.3	Nidogen 2	19.9	/
Dystroglycan	1.5	/	Periostin isoform 1 precursor	15.6	/
Elastin microfibril interfacer 1	42.5	/	Peroxidasin	6.4	/
Elastin microfibril interfacer 2	1.5	/	Prelamin-A/C	45.7	10.4
Elastin precursor	15.9	/	Proline and arginine rich end leucine rich repeat protein	23.9	/
Extracellular matrix protein 1	3.9	/	Serpin H1 precursor	43.1	2.1
Extracellular matrix protein 2	5.7	/	Serum albumin	28.5	9.4
Fast skeletal muscle troponin C	4.1	2.4	Tenascin	33.9	/
Fibrillin 2	117.9	/	Tenascin-X precursor	62.8	/
Fibrillin 3	32.7	/	Tropomyosin alpha-1 chain	15.0	10.2
Fibrillin-1	207.1	/	Tropomyosin alpha-3 chain	7.9	6.5
Fibromodulin	5.3	/	Trypsinogen precursor	34.9	19.6
Fibronectin 1	235.0	/	Tubulin beta chain	24.2	2.1
Fibronectin 1	229.8	/	Uncharacterized protein*	29.7	9.7
Fibronectin type III domain containing 3A	10.4	/	Uncharacterized protein**	10.6	2.3
Fibronectin type III domain containing 3B	15.7	/	Vimentin	56.0	22.4
Fibulin 2	7.5	/	Vitronectin	5.7	/

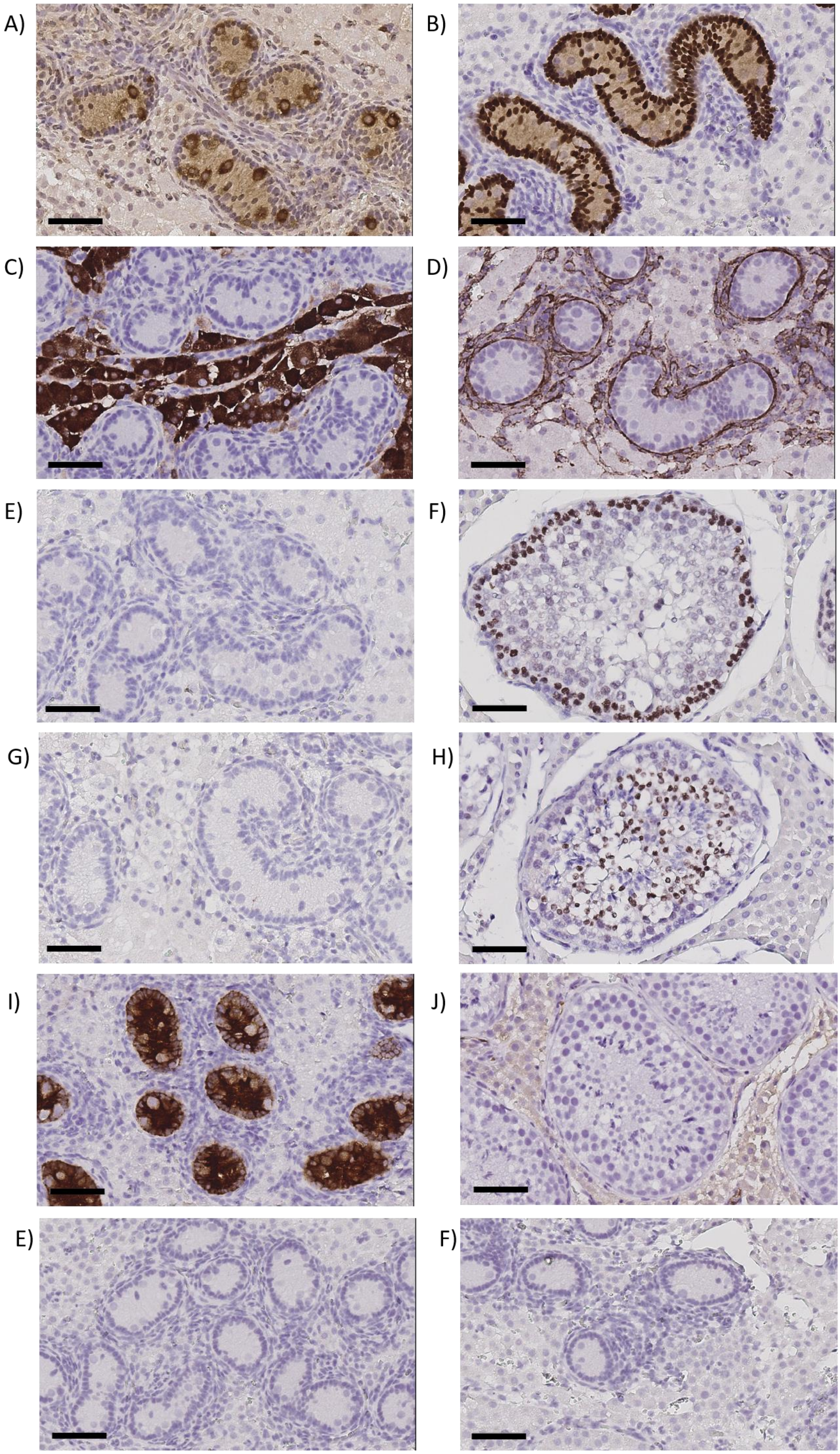
A)
B)
C)
D)
E)
F)
G)
H)
I)
J)
E)
F)

Figure S1. IHC controls. Porcine ITT recovered from pigs aged between 4 and 7 days old was used as positive control for DDX4 (A), SOX9 (B), CYP19A1 (C) and ACTA2 (D) immunostainings. Positive staining for SCP3 and CREM were detected in mature (6 months old) porcine testicular tissue (F-H) but not in ITT (E-G). The expression of AMH was detected in STs of porcine ITT (I) but not in mature testicular tissue (J). Pictures E and F represent negative controls (without primary antibodies) with anti-mouse (E) and anti-rabbit (F) secondary antibodies. Scale bars = 50 μm.
